# Gut Microbiota Modulation by Abelmoschus manihot (L.) Improves Circulating Metabolites and Alleviates Diabetic Nephropathy in db/db Mice

**DOI:** 10.1155/bmri/5630909

**Published:** 2026-10-08

**Authors:** Qing Xu, Yuhui Song, Hongmei Yu, Yurong Wang, Harvest F. Gu

**Affiliations:** ^1^ Laboratory of Molecular Medicine School of Basic Medicine and Clinical Pharmacy China Pharmaceutical University Nanjing Jiangsu Province China cpu.edu.cn

**Keywords:** *Abelmoschus manihot* (L.), diabetic nephropathy, intestinal microbiota, plasma metabolites

## Abstract

**Background:**

Huangkui capsule (HKC), derived from the ethanol extract of *Abelmoschus manihot* (L.) flowers, is widely used in China for treating kidney diseases, including diabetic nephropathy (DN). Our previous study demonstrated that HKC modulates the intestinal microbiota and circulating metabolites in non‐obese diabetic mice, a type 1 diabetes model. To further explore its efficacy, we evaluated HKC in *db/db* mice, a well‐established type 2 diabetes and DN model.

**Methods:**

An HKC cohort studied in 2022 was compared with historical Ctrl and DN cohorts studied in 2021. Shotgun metagenomic sequencing was performed to characterize intestinal microbiota changes, while liquid chromatography–mass spectrometry (LC–MS)‐based plasma metabolomics was used to identify alterations in circulating metabolites. The biological functions of the altered microbiota and plasma metabolites were analyzed, and the potential association between the intestinal microbiome and plasma metabolome was evaluated.

**Results:**

Compared with the historical DN cohort, the HKC cohort had higher abundances of *Streptococcaceae*, *Streptococcus*, and *Massilimaliae* and lower abundances of *Alloprevotella* and *Prevotellamassilia* in exploratory comparisons. In the HKC‐versus‐DN comparison, the archived gene set enrichment analysis reported 15 pathways with nominal positive enrichment. Additionally, 12 plasma metabolites were upregulated and 14 downregulated, including branched‐chain amino acids (DL‐leucine, DL‐valine, D‐isoleucine), organic acids (N‐methyl‐*α*‐aminoisobutyric acid, guanidineacetic acid), and choline.

**Conclusion:**

In *db/db* mice, the cohort receiving *A. manihot* (L.)‐derived HKC had lower urinary albumin‐to‐creatinine ratio (UACR) and different intestinal microbiota and plasma metabolite profiles than the historical DN cohort, highlighting its therapeutic potential for DN.

## 1. Introduction

Diabetes mellitus is a metabolic disease characterized by dysregulated glucose metabolism, primarily classified into type 1 diabetes (T1D), which can occur at any age but predominantly affects children and young adults, and type 2 diabetes (T2D), which accounts for approximately 90% of all cases [1, 2]. Both T1D and T2D can lead to chronic kidney disease (CKD), whereas T2D contributes to the largest proportion of the global diabetic kidney disease burden because of its substantially higher prevalence. Up to 40% of patients with T2D develop diabetic nephropathy (DN), the leading cause of end‐stage kidney disease (ESKD) [3, 4]. Dialysis and kidney transplantation remain the main treatment options for ESKD, but they impose significant burdens on patients, families, and healthcare systems [5]. Consequently, effective, mechanism‐based therapeutic strategies for DN are urgently needed to improve quality of life and reduce economic pressures.

Huangkui capsule (HKC), derived from the ethanol extract of *Abelmoschus manihot* (L.) flowers, is clinically used in China for treating kidney diseases, including DN [6]. Clinical studies and systematic reviews have demonstrated its efficacy and safety in managing primary glomerular disease and IgA nephropathy (IgAN) [7–9]. A recent multicenter, randomized, double‐blind, parallel, and controlled trial further confirmed that HKC combined with irbesartan reduced albuminuria in patients with T2D and DN [10]. However, HKC has not yet been incorporated into major international CKD or DN management guidelines, which currently emphasize evidence‐based interventions such as renin–angiotensin system blockade, sodium‐glucose cotransporter 2 inhibitors, glucagon‐like peptide‐1 receptor agonists, and nonsteroidal mineralocorticoid receptor antagonists [11–13]. Therefore, additional mechanistic and translational studies are needed to clarify the biological basis and potential clinical value of HKC in DN.

The intestinal microbiota, the body’s largest micro‐ecological system, plays a pivotal role in material and energy metabolism [14, 15]. Emerging evidence suggests that alterations in gut microbiota composition and circulating metabolites contribute to diabetes and DN by disrupting glucose homeostasis and immune regulation [16–19]. Our previous study revealed that HKC modulates intestinal microbiota and improves circulating metabolite levels in non‐obese diabetic (NOD) mice, a model of T1D and DN [20]. Our group previously evaluated HKC in *db/db* mice using complementary experimental designs. Yu et al. examined renal biomarkers located in the glomeruli and proximal and distal convoluted tubules, whereas a subsequent study evaluated HKC, irbesartan, and their combination using 16S rDNA sequencing, serum metabolomics, and kidney transcriptomics [21, 22]. The present study reused the previously reported processed Ctrl and DN shotgun metagenomic and plasma metabolomic datasets [23] and compared them with newly generated HKC data to obtain higher‐resolution taxonomic and functional profiles and examine their associations with renal phenotypes.

This study aimed to characterize differences in intestinal microbiota composition and circulating metabolites among the Ctrl, DN, and HKC cohorts in *db/db* mice, an established model for T2D and DN [24]. Unlike our previous work in NOD mice, this analysis focused on T2D–DN and integrated shotgun metagenomic sequencing, plasma metabolomics, renal phenotypic assessment, and microbiota–metabolite correlation analysis. We examined the associations among these measures to identify candidate links between intestinal microbiota, circulating metabolites, and renal phenotypes.

## 2. Methods

### 2.1. Animal Management and HKC Administration

All animal procedures were reviewed and approved by the Ethics Committee for Experimental Animals of China Pharmaceutical University (approval code: 2019‐08‐0003) and were conducted in accordance with institutional guidelines for the care and use of laboratory animals. Male diabetic *db/db* mice (BKS.Cg‐*Dock7^m^* +/+ *Lepr^db^*/J) and C57BL/6J mice (11 weeks old) were purchased from Changzhou Cavens Laboratory Animal Co., Ltd. (Changzhou, China). The animals were maintained in a specific pathogen‐free facility at China Pharmaceutical University (Xuanwu Campus) under a 12‐h light/dark cycle at 22^°^C ± 2^°^C and 50*%* ± 10*%* relative humidity, with *ad libitum* access to standard chow and water.

Body weight, urine volume, and blood glucose concentrations were measured weekly. C57BL/6J mice served as controls (Ctrl; *n* = 6), and eligible *db/db* mice constituted the DN group (initial *n* = 6). The HKC group consisted of a separate experimental cohort of eligible *db/db* mice (*n* = 6) studied using the same inclusion criteria and experimental procedures. One DN mouse died before terminal sampling and was excluded from all analyses reported here, yielding final group sizes of Ctrl, *n* = 6; DN, *n* = 5; and HKC, *n* = 6. Shotgun metagenomic sequencing used a subset of four animals per group. Cohort sizes were based on previous studies using *db/db* mice, the exploratory omics design, and the 3R principles. Mice in the HKC group received an aqueous HKC preparation (0.45 g/kg/day; Suzhong Pharmaceutical Group Co., Ltd., Taizhou, China) by oral gavage, whereas mice in the Ctrl and DN groups received an equal volume of distilled water. After 4 weeks of treatment, the mice were anesthetized, and peripheral blood was collected. The colon was ligated and harvested. The kidneys were perfused with phosphate‐buffered saline through an aortic catheter at 3 mL/min per kidney until blanched and then collected. Kidney specimens intended for histological analysis were fixed immediately, whereas the remaining tissue was stored at −80°C. The processed Ctrl and DN shotgun metagenomic and plasma metabolomic datasets were generated in 2021 and originally reported by Wu et al. [23]; these datasets were reused in the present study. The corresponding HKC datasets were generated in 2022 using the same service provider and analytical platforms. All surviving animals contributed to the physiological and histological analyses in Figure 1 and to plasma metabolomics in Figure 2 (Ctrl, *n* = 6; DN, *n* = 5; HKC, *n* = 6). Shotgun metagenomics and the paired microbiome–metabolome analyses in Figure 3 used the same subset of four animals per group; these samples are listed in Table S2 and are included in the metabolomics dataset in Table S3.

**Figure 1:**
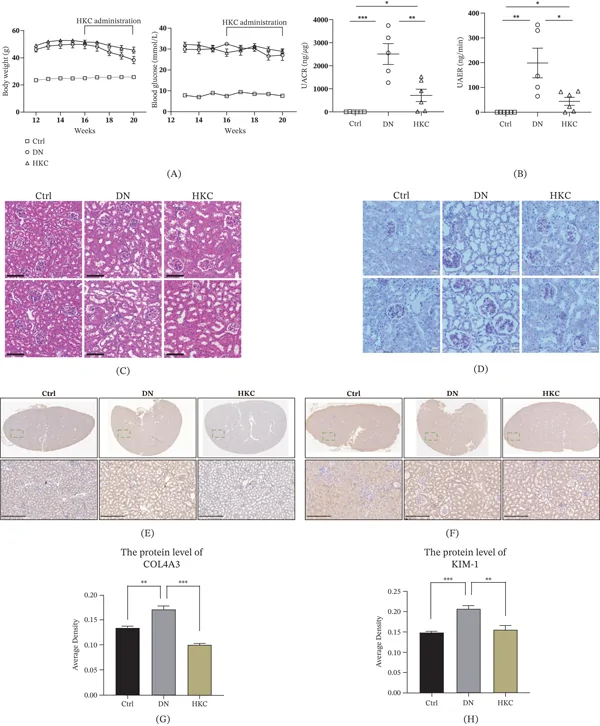
Physiological and histopathological measures across cohorts. (A) Body weight and blood glucose levels. (B) Urinary albumin‐to‐creatinine ratio (UACR) and urinary albumin excretion rate (UAER) at the end of the 4‐week administration period. (C) Images of kidney tissues of mice in the Ctrl, DN, and HKC groups stained with hematoxylin and eosin. Scale bars = 100 *μ*m. (D) Images of kidney tissues of mice in the Ctrl, DN, and HKC groups stained with periodic acid Schiff stain. Scale bars = 20 *μ*m. (E) Immunohistochemical detection of COL4A3 in randomly selected magnified fields of whole kidney sections. Scale bars = 200 *μ*m. (F) Immunohistochemical detection of KIM‐1 in randomly selected magnified fields of whole kidney sections. Brown DAB staining indicates immunopositivity for COL4A3 or KIM‐1. Scale bars = 100 *μ*m. (G, H) Average optical density values of COL4A3 and KIM‐1. Average density = integrated optical density/area. Data are presented as the mean ± SEM. Ctrl, *n* = 6; DN, *n* = 5; and HKC, *n* = 6.  ^∗∗∗^ *p* < 0.001,  ^∗∗^ *p* < 0.01,  ^∗^ *p* < 0.05. Blood‐glucose readings displayed as “HI” were above the meter’s upper measurement limit and were assigned 33.3 mmol/L for analysis and plotting.

Figure 2Plasma metabolomic differences among cohorts. (A) Descriptive PCA of plasma metabolomic profiles in the Ctrl, DN, and HKC cohorts. (B) Volcano plots of DEMs. The *x*‐axis represents log2FC (DN/Ctrl or HKC/DN), and the *y*‐axis represents ‐log10 of the nominal Welch *p* value. Dashed lines indicate the DEM cutoffs at log2FC = −0.25 and +0.25 and *p* = 0.05. DEMs were defined as nominal *p* < 0.05 from two‐sided Welch tests on log2‐transformed peak areas and |log2FC| > 0.25. Red and green indicate higher and lower abundance, respectively. (C) Venn diagram of the 130 DN‐versus‐Ctrl and 26 HKC‐versus‐DN DEMs, including 20 shared DEMs. (D) Heat map of the 20 shared DEMs; columns represent samples and rows represent metabolites. (E) Relative peak areas of 17 selected shared DEMs in the DN‐versus‐Ctrl and HKC‐versus‐historical‐DN comparisons; bars and error bars show the mean and SEM. (F) Metabolites that differed between the HKC and historical DN cohorts are highlighted in red. Red and blue arrows indicate higher and lower abundance, respectively; yellow labels indicate pathways. Dashed lines indicate omitted reactions or intermediates. Ctrl, *n* = 6; DN, *n* = 5; HKC, *n* = 6.  ^∗^ *p* < 0.05,  ^∗∗^ *p* < 0.01,  ^∗∗∗^ *p* < 0.001,  ^∗∗∗∗^ *p* < 0.0001 versus Ctrl; #*p* < 0.05, ##*p* < 0.01, ###*p* < 0.001 versus historical DN.

**Figure:**
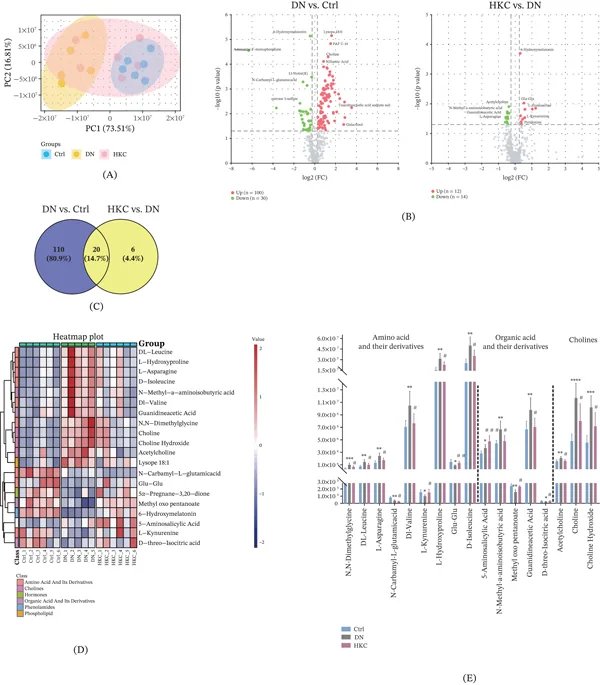

**Figure:**
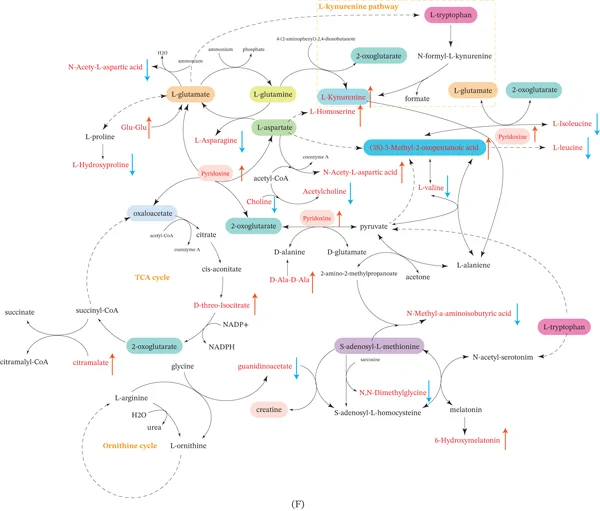

**Figure 3:**
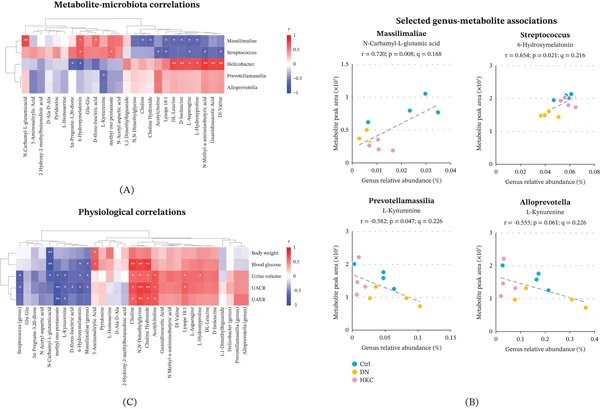
Correlations among plasma metabolites, intestinal microbiota, and physiological measures across cohorts. (A) Pearson correlations between 26 HKC‐versus‐DN DEMs and five bacterial genera. (B) Individual‐animal scatterplots for four key genera, each paired with the metabolite having the largest absolute r among the 26 candidates. Points represent Ctrl (blue), DN (yellow), and HKC (pink) animals. Dashed gray lines show pooled ordinary least‐squares fits. The displayed *q* values are adjusted over all 130 tests in A. (C) Correlations of UACR, UAER, urine volume, body weight, and blood glucose with the same 26 metabolites and five genera. Heatmaps use blue for negative and red for positive *r*, dendrograms show average‐linkage clustering. Analyses pooled 12 animals, four per cohort. Asterisks indicate two‐sided nominal *p* values:  ^∗^ *p* < 0.05,  ^∗∗^ *p* < 0.01. No correlations met BH‐adjusted *q* < 0.05 in A or C.

### 2.2. Hematoxylin and Eosin (H&E) and Periodic Acid–Schiff (PAS) Staining

Renal histology was assessed using a previously described procedure with minor modifications [21]. Kidney specimens collected after phosphate‐buffered saline perfusion were fixed in a formaldehyde‐based tissue fixative, processed routinely, and embedded in paraffin. The paraffin blocks were cut into 4‐*μ*m sections using a HistoCore BIOCUT microtome (Leica Biosystems, Germany). Sections were stained with H&E (Baso Diagnostics, Zhuhai, China) according to a standard protocol. For PAS staining, deparaffinized and rehydrated sections were oxidized with periodic acid, treated with Schiff reagent, and counterstained with hematoxylin. The stained sections were dehydrated, cleared, mounted, and examined using a CX23 bright‐field microscope (Olympus, Japan).

### 2.3. Immunohistochemical Staining and Digital Image Analysis

Paraffin‐embedded kidney sections were deparaffinized and rehydrated, followed by heat‐induced antigen retrieval in 10 mM citrate buffer (pH 6.0) for 20 min. After cooling, endogenous peroxidase activity was quenched and nonspecific binding was blocked with 10% normal goat serum. The sections were incubated overnight at 4°C with primary antibodies against collagen type IV *α*3 (COL4A3; 1:50, orb313870, Biorbyt) or kidney injury molecule 1 (KIM‐1/TIM‐1/HAVCR1; 1:100, NBP1‐76701, Novus Biologicals). After washing, the sections were incubated with an HRP‐based anti‐rabbit secondary detection reagent for 1 h at room temperature. Immunoreactivity was visualized with 3,3 ^′^‐diaminobenzidine (DAB; 1:20) for 30 s, and the nuclei were counterstained with hematoxylin. The sections were then dehydrated through graded ethanol, cleared, and coverslipped. Whole‐slide images were acquired in bright‐field mode using a VS200 slide scanner (Olympus, Tokyo, Japan). DAB‐positive staining was quantified using the Area Quantification algorithm in HALO software (version 2.0; Indica Labs, Corrales, NM, United States). Identical classifier settings and positivity thresholds were applied to all sections.

### 2.4. Microbial DNA Extraction and Sequencing

#### 2.4.1. DNA Extraction

Colon‐content samples were immediately frozen in liquid nitrogen and stored until analysis. Before extraction, the samples were rapidly thawed in a 25°C water bath. Total microbial genomic DNA was isolated using the E.Z.N.A. Stool DNA Kit (Omega Bio‐tek, Norcross, GA, United States) according to the manufacturer’s instructions. DNA quality and integrity were assessed by electrophoresis on a 1% agarose gel.

#### 2.4.2. Shotgun Metagenomic Sequencing

A separate aliquot of genomic DNA from each sample was fragmented using a Covaris M220 focused ultrasonicator (Covaris, Woburn, MA, United States). Metagenomic libraries with an average insert size of approximately 450 bp were prepared and sequenced in paired‐end mode on an Illumina high‐throughput sequencing system (Illumina, San Diego, CA, United States). Raw paired‐end reads were trimmed using Trimmomatic (version 0.38), and reads shorter than 70 bp after trimming were discarded. The remaining reads were assembled using a MEGAHIT (Version 1.2.9). CD‐HIT (Version 4.5.7) was used to generate nonredundant sequence clusters at 95% identity, and taxonomic classification was performed using Kraken2 (Version 2.0.6‐beta). Assignments outside Bacteria and Archaea were removed from the archived phylum‐, family‐, and genus‐level abundance matrices, and the remaining abundances were renormalized to 100% within each sample at each taxonomic level before downstream analyses.

### 2.5. Gene Set Enrichment Analysis (GSEA)

The archived GSEA used clusterProfiler to compare Kyoto Encyclopedia of Genes and Genomes (KEGG) pathway profiles between the Ctrl and DN cohorts and between the HKC and historical DN cohorts. The archived analysis ranked genes by log2 fold change and evaluated enrichment against the KEGG database. Pathways with an absolute normalized enrichment score > 1 and nominal *p* < 0.05 were considered nominally enriched. Benjamini–Hochberg (BH)‐adjusted *q* values were calculated separately within each pairwise contrast, with *q* < 0.05 indicating FDR‐controlled enrichment.

### 2.6. Metabolomic Analysis of Plasma Samples

For each sample, 100 *μ*L of plasma was mixed with 400 *μ*L of methanol, vortexed, incubated on ice for 5 min, and centrifuged at 15,000 ×*g* and 4°C for 5 min. The supernatant was mixed with one‐half volume of MS‐grade water and centrifuged again at 15,000 ×*g* and 4°C for 20 min. The final supernatant was used for LC–MS analysis. A blank consisting of 53% methanol containing 0.1% formic acid was processed in parallel.

Chromatographic separation was performed using an ExionLC AD system coupled to a QTRAP 6500+ mass spectrometer. Positive‐ion analysis used a BEH C8 column (100 × 2.1 mm, 1.9 *μ*m) at 0.35 mL/min with water containing 0.1% formic acid (mobile phase A) and acetonitrile containing 0.1% formic acid (B): 5% B for 1 min, 5%–100% B over 1–24 min, 100% B to 28 min, return to 5% B at 28.1 min, and equilibration to 30 min. Negative‐ion analysis used an HSS T3 column (100 × 2.1 mm) with the same mobile phases and flow rate: 2% B for 1 min, 2%–100% B over 1–18 min, 100% B to 22 min, return to 5% B at 22.1 min, and equilibration to 25 min. Source settings were curtain gas, 35 psi; collision gas, medium; temperature, 500°C; ion‐source gases 1 and 2, 55 psi; and ion‐spray voltage, +5500 V or −4500 V for positive‐ or negative‐ion mode, respectively.

Peak integration and correction were performed using SCIEX OS Version 1.4. Metabolite features were annotated by the commercial service provider using its established MRM‐based workflow and reference library. The QTRAP 6500+ was operated in unit‐resolution MRM mode; high‐resolution accurate‐mass and mass‐error data were not generated. The minimum peak height was 500, Gaussian smoothing width was 1, and signal‐to‐noise threshold was 5. Integrated peak areas were used as measures of relative metabolite abundance. Principal component analysis included all 448 annotated metabolite features after preprocessing. Metabolite peak areas were log2‐transformed for statistical testing; log2FC was calculated from the ratio of group mean peak areas on the original scale (DN/Ctrl or HKC/DN). For each pairwise comparison, two‐sided Welch’s *t*‐tests were performed on log2‐transformed peak areas for all 448 features. Metabolite features with nominal *p* < 0.05 and |log2FC| > 0.25 were considered differentially abundant metabolites (DEMs); this exploratory screening did not use an FDR or VIP cutoff. Sample‐level peak areas, log2FC, Welch *p* values, and DEM assignments are provided in Table S3.

### 2.7. Statistical Analysis

Statistical analyses were performed using IBM SPSS Statistics Version 23.0 and R Version 3.6.0, and figures were generated using GraphPad Prism Version 8.0. Physiological and histological endpoints were analyzed using Student’s *t*‐test or the Wilcoxon rank‐sum test, as appropriate. Metabolomic comparisons were performed using two‐sided Welch’s *t*‐tests after log2 transformation of peak‐area data. Data are presented as the mean ± standard error of the mean (SEM) unless otherwise stated. Blood‐glucose readings displayed as “HI” exceeded the meter’s upper measurement limit (> 33.3 mmol/L) and were assigned a value of 33.3 mmol/L for statistical analysis and graphical presentation.

Principal component analysis in Figure 4B was performed on centered, unscaled genus‐level relative abundances and interpreted descriptively. The ellipses were used only to visualize within‐group dispersion. For Figure 4C–G, HKC and historical DN abundances were compared using two‐sided Student’s *t*‐tests on the renormalized relative‐abundance data. The reported *p* values are nominal and unadjusted. Differential metabolite screening used the nominal *p* value and fold‐change thresholds specified in Section 2.6. BH adjustment was applied separately within each pairwise contrast to the differential KEGG ortholog and gene‐set enrichment analyses; these adjusted results are reported as *q* values, with *q* < 0.05 indicating FDR‐controlled significance. Throughout the manuscript, *p* denotes a nominal *p* value and *q* a BH‐adjusted *p* value. Metabolic pathways were annotated using the KEGG database and visualized using MetaboAnalyst 5.0 [25].

**Figure 4:**
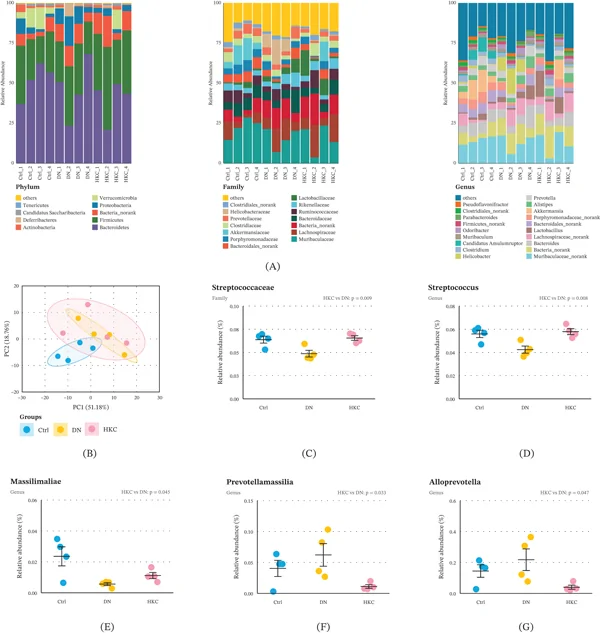
Intestinal microbiota composition across cohorts. (A) Relative abundances of the top‐ranked phyla (left), families (middle), and genera (right). Only bacterial and archaeal assignments were included; the remaining prokaryotic taxa were pooled as “others” after within‐sample renormalization to 100%. (B) Descriptive genus‐level PCA of the Ctrl, DN, and HKC cohorts. PC1 and PC2 explained 51.18% and 18.76% of the variance, respectively. Ellipses visualize within‐group dispersion and do not represent significance tests. (C‐G) Relative abundances of *Streptococcaceae* (family), *Streptococcus*, *Massilimaliae*, *Prevotellamassilia*, and *Alloprevotella* (genera), respectively. Points show individual animals, and horizontal lines and error bars show the mean and SEM. Nominal unadjusted *p* values are from two‐sided Student’s *t*‐tests comparing HKC with the historical DN cohort (*n* = 4 per group).

For Figure 3, Pearson correlations were calculated using renormalized genus‐level abundances, untransformed metabolite peak areas, and physiological measures. Body weight and blood glucose were taken at Week 20. The analysis included five genera and the 26 DEMs selected from the full HKC‐versus‐DN metabolomics comparison. Heatmaps used average‐linkage clustering with Euclidean distances between correlation profiles. BH correction was applied separately to the 130 genus‐metabolite tests in Figure 3A and the 155 physiological correlations in Figure 3C.

## 3. Results

### 3.1. Physiological Indices and Histopathological Analysis

Body weight, blood glucose, and UACR were measured in the Ctrl, DN, and HKC cohorts. UACR was higher in the DN cohort than in the Ctrl cohort, and the HKC cohort had lower UACR than the historical DN cohort at the 4‐week assessment (Figure 1A,B; Table S1). Body‐weight trajectories are shown in Figure 1A. Several blood‐glucose readings exceeded the meter’s upper measurement limit (> 33.3 mmol/L). These readings were entered as 33.3 mmol/L, so blood‐glucose comparisons were interpreted descriptively. Representative H&E and PAS images showed qualitative differences in glomerular and tubular morphology among the cohorts (Figure 1C,D). COL4A3 and KIM‐1 immunoreactivity was higher in the DN cohort than in the Ctrl cohort; values in the HKC cohort were lower than in the historical DN cohort and were descriptively closer to the Ctrl cohort (Figure 1E–H).

### 3.2. Alterations to the Composition of the Intestinal Microbiota

Shotgun metagenomic sequencing was used to characterize intestinal microbiota composition in the Ctrl, DN, and HKC cohorts using the filtered, renormalized phylum‐, family‐, and genus‐level matrices (Figure 4A; Table S2). Genus‐level PCA showed partial overlap among the three groups; PC1 and PC2 explained 51.18% and 18.76% of the variance, respectively (Figure 4B). The PCA plot was interpreted descriptively and did not test statistical separation among groups. In exploratory HKC‐versus‐historical‐DN comparisons, *Streptococcaceae* (family; *p* = 0.009), *Streptococcus* (genus; *p* = 0.008), and *Massilimaliae* (genus; *p* = 0.045) were more abundant in the HKC cohort, whereas *Prevotellamassilia* (genus; *p* = 0.033) and *Alloprevotella* (genus; *p* = 0.047) were less abundant (Figure 4C–G). For these selected taxa, the HKC‐versus‐DN differences were generally in the same direction as the Ctrl‐versus‐DN differences.

### 3.3. GSEA of the Altered Intestinal Microbiota

The archived GSEA reported 25 nominally enriched KEGG pathways in the HKC‐versus‐historical‐DN comparison (absolute normalized enrichment score > 1 and nominal *p* < 0.05), of which 15 showed positive enrichment. BH‐adjusted *q* values were also calculated, with *q* < 0.05 indicating FDR‐controlled enrichment. Fourteen pathways were nominally enriched in both the HKC‐versus‐DN and Ctrl‐versus‐DN comparisons (Figure 5A,B). Based on enrichment scores, the two pathways with the largest positive and negative scores were identified in each comparison (Figure 5C,D). Figure 5E,F shows gene‐level log2 fold‐change distributions within selected pathways. In the Ctrl‐versus‐DN comparison, the largest positive enrichment scores were observed for “cationic antimicrobial peptide (CAMP) resistance” (ES = 0.80) and “biofilm formation‐*Escherichia coli*” (ES = 0.76), whereas the largest negative scores were observed for “prenyltransferases” (ES = −0.74) and “steroid degradation” (ES = −0.67) (Figure 5C). In the HKC‐versus‐DN comparison, the largest positive scores were observed for “bacterial chemotaxis” (ES = 0.78) and “flagellar assembly” (ES = 0.64), whereas the largest negative scores were observed for “prenyltransferases” (ES = −0.78) and “chlorocyclohexane and chlorobenzene degradation” (ES = −0.77) (Figure 5D). The nominally enriched pathways were mainly associated with bacterial defense, nutrient transport, and motility.

Figure 5Functional differences in the intestinal microbiota across cohorts. (A, B) GSEA of KEGG pathways for the Ctrl‐versus‐DN and HKC‐versus‐historical‐DN comparisons. Red outlines identify selected pathways shared between the comparisons, not enrichment direction. The archived analysis reported 15 pathways with positive enrichment in the HKC‐versus‐DN comparison and 14 nominally enriched pathways shared between comparisons. Dot color indicates the nominal *p* value, dot size indicates the number of genes in the pathway, and gene ratio is the proportion of genes contributing to the enrichment score. Nominal enrichment was defined as an absolute normalized enrichment score > 1 and *p* < 0.05. (C, D) GSEA plots for the two pathways with the largest positive and negative enrichment scores in each comparison. (E, F) Distributions of gene‐level log2 fold changes within selected pathways for the Ctrl‐versus‐DN and HKC‐versus‐DN comparisons. Panels E and F display pathways with BH‐adjusted *q* < 0.05.

**Figure:**
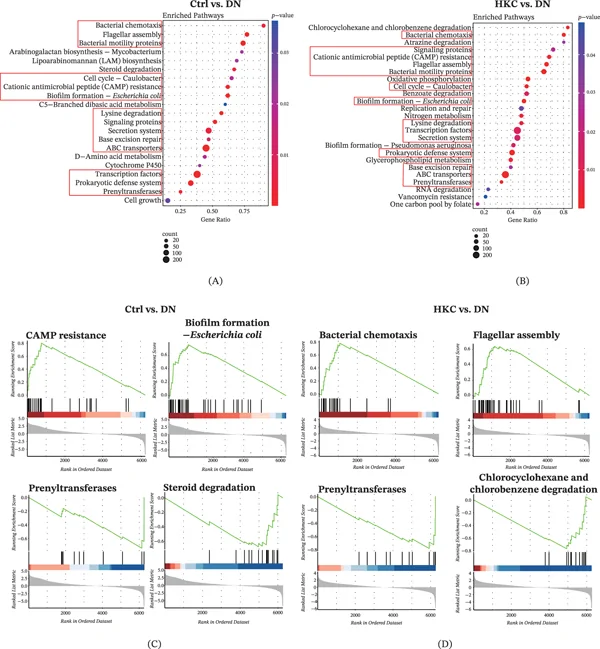

**Figure:**
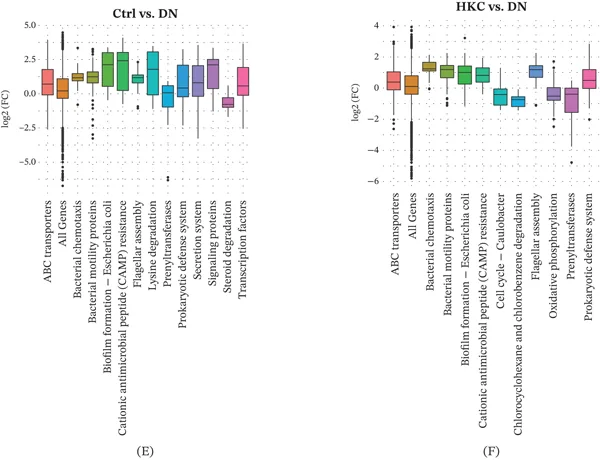

### 3.4. Plasma Metabolite Differences Among Cohorts

Plasma metabolomic profiling detected 448 annotated metabolite features in each group (Table S3). In the descriptive PCA, Ctrl and DN samples occupied different regions, while HKC samples overlapped both cohorts (Figure 2A). Using nominal *p* < 0.05 from Welch tests on log2‐transformed peak areas and |log2FC| > 0.25, 130 DEMs were identified between the Ctrl and DN groups and 26 between the historical DN and HKC groups, including 20 shared DEMs (Figure 2C). Volcano plots showed that 100 DEMs were higher and 30 were lower in the DN group than in the Ctrl group, whereas 12 were higher and 14 were lower in the HKC group than in the historical DN group (Figure 2B). A heat map was generated for the 20 DEMs shared between the two comparisons (Figure 2D). For many of these shared DEMs, the HKC‐versus‐DN difference was in the direction opposite to the DN‐versus‐Ctrl difference. Of the 20 shared DEMs, 17 are shown in Figure 2E: nine amino acids and related derivatives, five organic acids and related derivatives, and three choline‐related compounds.

A metabolic pathway map was generated for DEMs that differed between the HKC and historical DN cohorts to describe associated pathways and potential functions (Figure 2F). These DEMs mapped mainly to amino acid production and degradation and included L‐kynurenine and pyridoxine. Their abundance patterns involved metabolites connected to the *γ*‐glutamyl, tricarboxylic acid, and methionine cycles; these pathway annotations are descriptive and do not demonstrate that HKC altered downstream synthesis or metabolism.

### 3.5. Metabolite Set Enrichment and Network Analysis

To gain further insight into biological functions associated with the DEMs, 26 DEMs between the historical DN and HKC cohorts were selected for metabolite set enrichment and network analyses. Enrichment was observed for the pathways “valine, leucine, and isoleucine biosynthesis”, “aminoacyl‐tRNA biosynthesis”, “glycine, serine, and threonine metabolism”, and “glycerophospholipid metabolism”. Of these, the pathway with the strongest enrichment signal was “valine, leucine, and isoleucine biosynthesis” (Figure 6A). In addition, the DEMs between the historical DN and HKC cohorts were associated with the pathways “glycine, serine, and threonine metabolism” and “glycerophospholipid metabolism” (Figure 6B). Meanwhile, DEMs between the historical DN and HKC cohorts were included in metabolite–disease interaction network analysis. Overall, 12 circulating metabolites that differed between the HKC and historical DN cohorts mapped to neurological disorders (e.g., Alzheimer’s disease and schizophrenia) and inherited disorders (e.g., maple syrup urine disease, dihydrolipoamide dehydrogenase deficiency, branched‐chain keto acid dehydrogenase kinase deficiency, and lipoyltransferase‐1 deficiency) (Figure 6C). In addition, metabolite–metabolite interaction network analysis was performed. The functional relationships of the identified DEMs between the historical DN and HKC cohorts are presented in Figure 6D.

**Figure 6:**
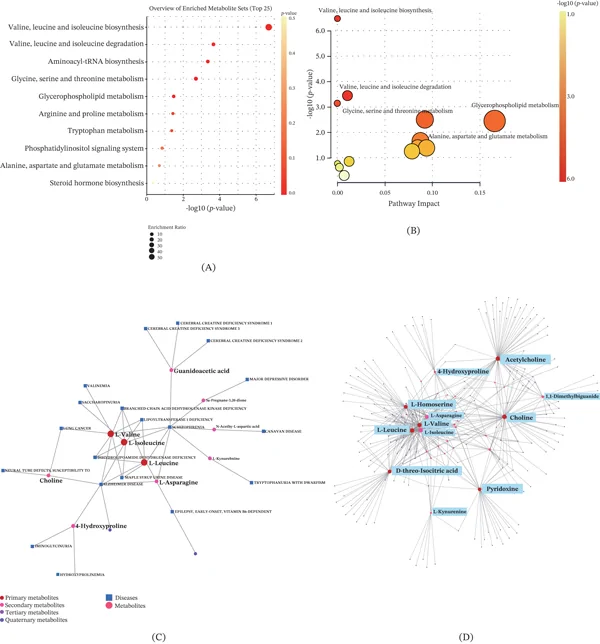
Pathway and interaction–network analyses of plasma DEMs. (A) Enriched pathways of the 26 DEMs between the HKC and historical DN cohorts. (B) Pathway analysis of the same DEMs. Color represents the pathway ‐log10(p) value. The node radius is based on the pathway impact values. (C) Metabolite–disease interaction network of the DEMs between the historical DN and HKC cohorts. (D) Integrated network of the key DEMs based on the KEGG database.

### 3.6. Correlation Analysis of the Altered Plasma Metabolites, Intestinal Microbiome, and Physiological Parameters

Across the 12 paired animals, 27 of 130 genus–metabolite correlations had nominal *p* < 0.05 (Figure 3A). The four selected associations were *Massilimaliae* with N‐carbamyl‐L‐glutamic acid (*r* = 0.720, *p* = 0.008), *Streptococcus* with 6‐hydroxymelatonin (*r* = 0.654, *p* = 0.021), and *Prevotellamassilia* and *Alloprevotella* with L‐kynurenine (*r* = −0.582, *p* = 0.047 and *r* = −0.555, *p* = 0.061, respectively; Figure 3B). Their BH‐adjusted *q* values were 0.168, 0.216, 0.226, and 0.226, respectively. In Figure 3C, 38 of 155 physiological correlations had nominal *p* < 0.05. UACR was negatively correlated with *Streptococcus* (*r* = −0.630, *p* = 0.028) and *Massilimaliae* (*r* = −0.599, *p* = 0.040). No correlations in either heat map remained significant at *q* < 0.05 after BH correction.

## 4. Discussion

In the present study, shotgun metagenomic sequencing was used to compare intestinal microbiota profiles across the Ctrl, DN, and HKC cohorts. These comparisons characterize microbial patterns and their associations with DN‐related phenotypes but do not isolate an HKC treatment effect [23]. The HKC cohort had higher abundances of *Streptococcaceae*, *Streptococcus*, and *Massilimaliae* and lower abundances of *Alloprevotella* and *Prevotellamassilia*. These findings are consistent with previous reports linking gut microbial alterations to kidney disease and metabolic disorders. For example, altered gut microbial composition has been reported in patients with IgAN [26], and compositional shifts in gut microbiota have also been observed in patients with T2D–DN [27]. However, the intestinal microbiota of *db/db* mice differs from that of NOD mice [20]. T2D is a noncommunicable metabolic syndrome characterized by pancreatic beta‐cell dysfunction and insulin resistance, whereas T1D is a chronic autoimmune disease characterized by insulin deficiency and the resultant hyperglycemia [28, 29]. Mendelian randomization analysis of genome‐wide association studies of DN indicated that potential causal relationships between gut microbiota and DN may differ between T1D and T2D and across different stages of DN [30].

The archived functional analysis reported 14 nominally enriched pathways shared between the Ctrl‐versus‐DN and HKC‐versus‐DN comparisons. The enriched pathways in the HKC group were mainly related to bacterial defense, motility, and biosynthesis. For example, some bacterial adenosine triphosphate‐binding cassette transporters have been investigated as vaccine and therapeutic targets [31]. Flagellin is a significant component of the flagellar assembly pathway that stimulates innate and mucosal immunity [32]. Chemotaxis and flagellar assembly are involved in bacterial motility and responses to external stimuli [33]. Other pathways included “CAMP resistance,” “prokaryotic defense system”, and “biofilm formation‐*Escherichia coli*.” These functional differences may reflect variation in microbial survival, movement, and colonization across cohorts.

Several circulating metabolites identified in this study, including acetylcholine, L‐asparagine, L‐hydroxyproline, and choline, are related to renal vascular regulation, amino acid and glucose metabolism, collagen turnover, and oxidative/metabolic stress, which are processes implicated in T2D and DN [34, 35]. The pathways associated with DEMs between the historical DN and HKC cohorts were primarily related to amino acid degradation or processing. Changes in amino acid metabolism can lead to dysregulation of renal filtration, and several amino acid‐related metabolites have been proposed as diagnostic biomarkers of early‐stage DN [36]. In agreement with a previous study of NOD mice conducted by our group [20], the present study found that relative plasma abundances of the branched‐chain amino acids (BCAAs) DL‐leucine, DL‐valine, and D‐isoleucine were higher in the DN cohort than in the Ctrl cohort, whereas the HKC‐versus‐historical‐DN differences were in the opposite direction. Conversely, (3S)‐3‐methyl‐2‐oxopentanoic acid and pyridoxine were higher in the HKC cohort than in the historical DN cohort. BCAAs are obtained from the diet [37] and have been associated with T2D and gestational diabetes [38]. Elevated BCAA levels have been linked to altered BCAA metabolism and mechanistic target of rapamycin complex 1 signaling in obesity and insulin resistance [39, 40]. *Streptococcus* and *Massilimaliae* also showed negative correlations with several BCAA features in Figure 3A, linking these cohort‐level patterns descriptively.

Various DEMs, such as D‐isoleucine, DL‐leucine, and L‐asparagine, were identified in the plasma of *db/db* mice with potentially strong associations with DN [41, 42]. L‐asparagine and L‐hydroxyproline are associated with incident diabetes [43]. Guanidineacetic acid, primarily synthesized in kidneys and pancreas, is depleted in both patients with diabetes and CKD [44], whereas the kynurenine/tryptophan ratio has been associated with albuminuria and responsiveness to angiotensin receptor blocker therapy in patients with DKD [45]. Moreover, 6‐hydroxymelatonin and L‐kynurenine are linked to tryptophan/melatonin metabolism, glucose homeostasis, and energy metabolism [46, 47]. In Figure 3B, *Streptococcus* was positively correlated with 6‐hydroxymelatonin, whereas *Prevotellamassilia* and *Alloprevotella* were negatively correlated with L‐kynurenine. These associations identify candidate links to tryptophan‐related metabolism for further study.

The HKC and control cohorts were nonconcurrent, so treatment status was confounded with study year and potential batch effects. Filtering the taxonomic matrices could not exclude residual sequence misclassification or revalidate the archived functional analysis in Figure 5. HKC is clinically used in China and has shown albuminuria‐lowering effects in a randomized clinical study of patients with T2D and diabetic kidney disease [10]. Nevertheless, major international guidelines for CKD and diabetes‐related CKD do not currently recommend HKC as a standard therapy [11–13]. The present findings therefore generate mechanistic hypotheses for further investigation rather than direct clinical evidence for guideline‐level implementation. Future human studies should evaluate whether HKC modifies gut microbiota and circulating metabolites in patients with T2D and DN and whether these changes are associated with clinically meaningful outcomes, such as albuminuria reduction, estimated glomerular filtration rate preservation, and inflammatory or metabolic biomarkers.

## 5. Conclusion

In this exploratory study, the HKC cohort differed from the historical DN cohort in intestinal microbiota composition and plasma metabolite profiles and had lower UACR and lower COL4A3 and KIM‐1 immunoreactivity. Several microbiota and metabolite measures were correlated, suggesting that *A. manihot* (L.) may exert therapeutic effects on DN through the gut–kidney axis and supporting further mechanistic and translational validation of HKC in DN.

## Author Contributions

Q.X. and Y.S. performed the experiments and analyzed data. H.Y. and Y.W. evaluated the data. Q.X. and Y.S. wrote the original draft. H.F.G. conceptualized the study, reviewed, and edited the manuscript. Q.X. and Y.S. contributed equally to this work.

## Funding

This study was supported by the Cooperation Research Project (CPU20200228), funded by Suzhong Pharmaceutical Group Co. Ltd., the manufacturer of Huangkui capsule.

## Disclosure

The authors of the manuscript have read and agreed to the consent for publication. All authors approved the final version of the manuscript. The funder had no role in the study design, data collection, data analysis, interpretation of the results, manuscript preparation, or the decision to submit the manuscript for publication.

## Conflicts of Interest

The authors declare no conflicts of interest.

## Supporting Information

Additional supporting information can be found online in the Supporting Information section.

## Supporting information


**Supporting Information 1**  

**Supporting Information 2**  

**Supporting Information 3**  

## Data Availability

The data supporting the findings of this study are provided within the article and its Supporting Information. The processed Ctrl and DN shotgun metagenomic and metabolomic datasets were originally reported by Wu et al. [23] and were reused in the present study for a secondary comparative analysis with the HKC group. The processed sample‐level taxonomic abundance matrix and plasma metabolomic data for all three groups are provided in the Supporting Information.
